# Ultra-conserved RNA: a novel biological tool with diagnostic and therapeutic potential

**DOI:** 10.1007/s12672-023-00650-1

**Published:** 2023-04-10

**Authors:** Tingye Wang, Feng Li, Zhanping Lu

**Affiliations:** 1 Department of Basic Medicine and Medical Technology, Medical College, Yangzhou University, Yangzhou, Jiangsu China; 2 Jiangsu Key Laboratory of Experimental & Translational Non-Coding RNA Research, Yangzhou, Jiangsu China

**Keywords:** Ultra-conserved RNA, Non-coding RNA, MicroRNA, Cancer, Regulate

## Abstract

Ultra-conserved RNA (ucRNA) is a subset of long non-coding RNA, that is highly conserved among mice, rats and humans. UcRNA has attracted extensive attention in recent years for its potential biological significance in normal physiological function and diseases. However, due to the instability of RNA and the technical limitation, the function and mechanism of ucRNAs are largely unknown. Over the last two decades, researchers have made a lot of efforts to try to lift the veil of ucRNA in nervous, cardiovascular system and other systems as well as cancers. Since the concept of the glymphatic system is relatively new, we summarized here recent findings on the functions, regulation and the underlying mechanisms of ucRNAs in physiology and pathology. Meanwhile, pathology in some diseases is likely to contribute to abnormal expression of ucRNA in turn. We also discuss the technical challenges and bright prospects for future applications of ucRNAs in the diagnosis and treatment of diseases.

## Introduction

Ultra-conserved RNAs (ucRNAs) are transcribed from 481 conserved DNA fragments present in the genome. They represent a subset of long non-coding RNAs (lncRNAs), which are non-coding RNAs over 200 nucleotides in length. UcRNAs were first discovered in 2004 by Gill Bejerano et al., and are highly conserved among mice, rats, and humans [1].

UcRNAs are highly conserved not only among species, but also throughout evolution. The presence of evolutionary conservation indicates ucRNAs may have critical physiological functions. In addition, several studies have shown they play an important role in regulating gene expression during carcinogenesis and injury, which are considered to be low conserved or tissue-specific biological processes. However, many of the specific mechanisms underlying ucRNA function remain incompletely understood. Therefore, further research of ucRNAs is warranted to deepen understanding of the physiological and pathological significance of these molecules.

Past studies have demonstrated the expression levels of ucRNAs impact normal human development. Alterations in expression levels play a significant role in the development of various diseases, including heart defects, tuberculosis, and nerve pain, but also can be exploited to treat these disease states. In addition, studies have shown that many ucRNAs can play either a positive or negative regulatory role in cancer progression. For example, aberrantly expressed uc.63 + can act as a carcinogen to promote cancer cell invasion and migration in a variety of tumor tissues. Most research on ucRNAs thus far has focused on their important roles in cancer, and has uncovered their potential as diagnostic markers and therapeutic targets. This paper reviews the current scientific understanding of ucRNAs in human physiological development and pathological disease development, providing an important reference for further ucRNA research.

## Biogenesis and functional mechanisms of ultra-conserved RNA

UcRNAs are transcripts from ultra-conserved regions (UCRs) which are widely transcribed across tissues. Of the 481 UCRs, 111 are designated as partly-exonics (E), which overlap with the known mRNAs of human protein-coding genes, thus exhibiting significant functions in RNA processing specificity. 256 non-exonics (N), which have no evidence of encoding proteins, play an important role in transcriptional regulation at the DNA level. For the remaining 114, the evidence for transcription is inconclusive and we called these possibly exonics (P).

Through microarray analysis, about 93% of the UCRs in the 19 specimens are expressed by background in at least one sample, we believe these as T-UCRs. The transcription frequencies of the three different types of UCRs (E, P, N) are similar. Of all the normal tissues analyzed, 84 of the 962 UCRs are transcribed bidirectionally, while 241 are transcribed from only one strand. In addition, strand-specific qRT-PCR and microarray analysis showed that about 83% of non-exonic T-UCRs are not intron transcripts of long precursor transcripts of known host genes, but truly independent non-coding transcripts [2].

Multiple expression techniques, including northern blot, qRT-PCR, and genome-wide microarray analysis, demonstrated that UCRs have distinct transcriptional signatures at vulnerable sites in the genome and cancer-associated genomic regions (CAGRs). This strong association between T-UCRs and predefined regions in the cancer genome suggests that these areas can be used as both therapeutic markers and targets [2]. T-UCR has been shown to drive malignant transformation of human diseases by regulating non-coding RNAs and/or protein-coding genes.

As a calss of long non-coding RNA, ucRNAs regulate target gene expression through multiple mechanisms. One proposed mechanism is that ucRNAs act as competitive endogenous RNAs (ceRNAs), which compete for binding to microRNAs (miRNAs), resulting in the stabilization of mRNA. In vitro, UCRs were cloned in luciferase reporter vectors to assess the possible direct interaction with miRNA, except that uc.348(N) did not interact with miR-155 as indicated by the luciferase assay. In vivo, the results were consistent. In addition, 87 miRNAs were found to be negatively correlated with T-UCRs expression levels [2]. Another proposed mechanism for ucRNA function is through the regulation of alternative splicing. Alternative splicing is a process by which different mRNA transcripts can be produced from the same gene, leading to the production of different protein isoforms. It has been suggested that ucRNAs may play a role in regulating alternative splicing by binding to splicing factors or other RNA-binding proteins [3]. UcRNAs may also function as scaffolds for the assembly of protein complexes involved in gene regulation [4]. For example, some ucRNAs have been shown to bind to both DNA and RNA-binding proteins, suggesting that they may play a role in the regulation of gene expression at the transcriptional or post-transcriptional level.

## Physiology

**Fig. 1:**
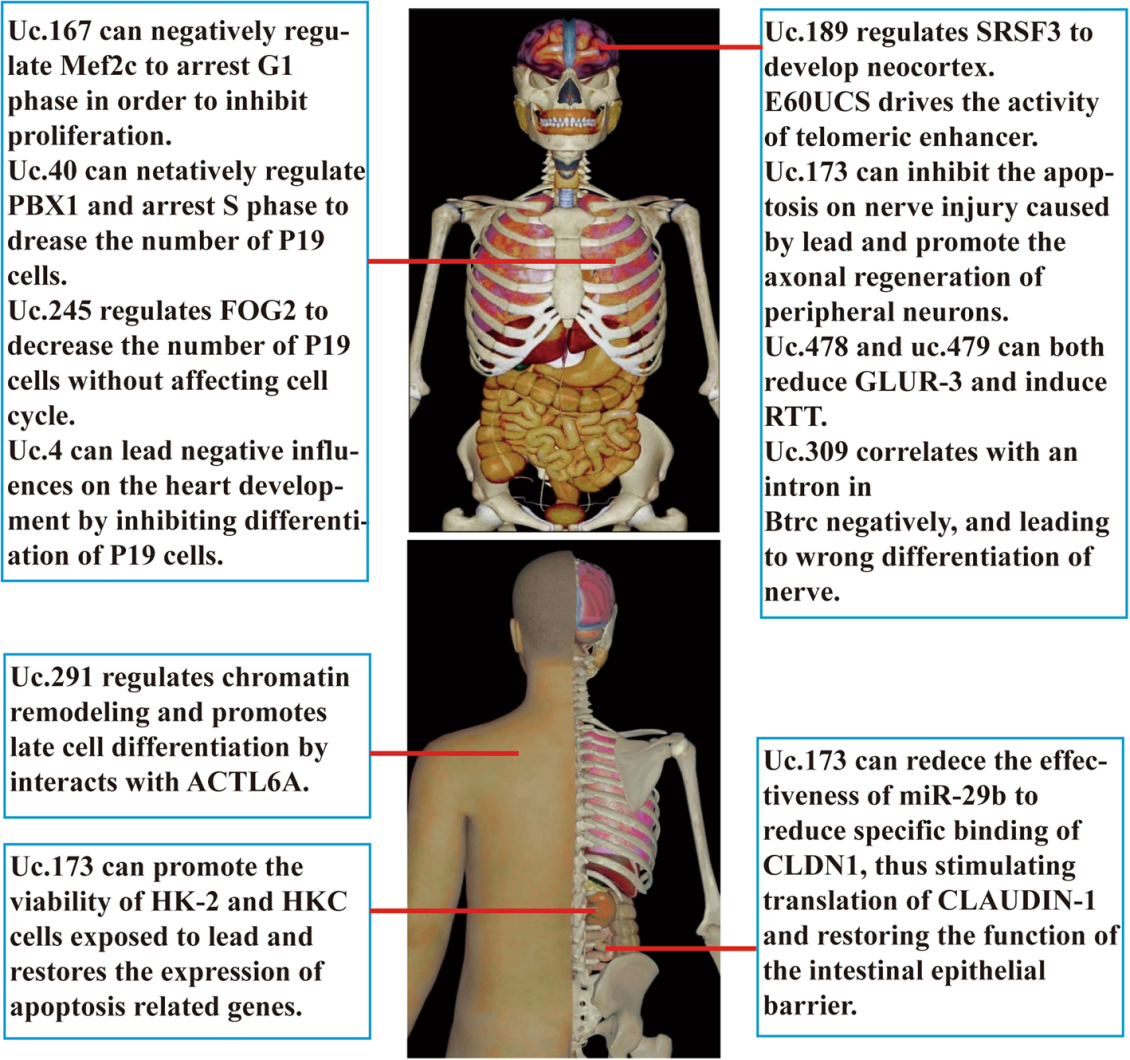
The roles and potential mechanisms of ucRNAs in physiological activities. Influences of each ucRNA are shown on the normal physiological activities of various systems and organs of the human body, mainly including heart, nerves, skin, kidney and intestine. At the same time, this figure also indicates the mechanisms of ucRNA to some extent

Normal physiological activities of biomolecules are critical for maintaining human health and development throughout life. Some ucRNAs have been found to influence physiological activities and the maintenance of physiological states (as shown in Fig. 1).

### Neuroendocrine system

The nervous system controls the activities of various organs and systems, while the endocrine system is the most important metabolic regulator in the body. These body systems work together closely to modulate a wide variety of physiological functions. The expression of ucRNAs in these systems plays a critical role in both neuroendocrine development and regulation of neuroendocrine physiological activity.

It has been reported that the ucRNA, uc.189, promotes the development of the neocortex by limiting its expression to the ventricular zone (VZ) which is located in the nucleus. It was also identified the ucRNA, uc.189, can upregulate its regulatory target serine-arginine-rich splicing factor 3 (SRSF3) protein expression, which prevents the abnormal proliferation of neural progenitor cells [5]. E60UCS is important for brain development. It is transcribed from a 25 kb fragment downstream of the Pax6 3’ UTR in the large final intron of the adjacent *Rlp4* gene. This ucRNA is mainly expressed in the optic cup, optic fold, telencephalon, diencephalon, and the precursor P3 peptide. E60UCS drives telencephalon enhancer activity by cooperating or hierarchizing with the *Pax6* surrounding it and its expression remains unchanged throughout development [6]. The level of uc.173 was found to decrease significantly in the established mouse model representing lead induced nerve injury. They demonstrated uc.173 plays an anti-apoptotic role in lead induced nerve injury at multiple levels. MiR-291a-3p, a target gene of uc.173, has apoptosis-associated target genes (*PI3K*, *NIK* and *Cn*) and a DNA-damage repair-associated target gene (*RAD23B*). Therefore, they hypothesized that uc.173 may mediate apoptotic inhibition through miR-291a-3p, which has not been proved [7]. In another study, Wu found that uc.173 is highly upregulated in the prefrontal cortex of Sprague–Dawley (SD) rats after exposure to methylphenidate and may regulate neuronal differentiation of rat pheochromocytoma cells. They also found uc.173 and ubiquitin-conjugating enzyme E2B (UBE2B) have sensory overlap and thus participate in axonal regeneration of peripheral neurons [8]. Furthermore, it has been shown MeCP2 directly regulates uc.478 and uc.479 to influence the function of receptor and protein stability, thus decreasing GLUR-3 protein level, which induces the development of Rett syndrome (RTT) progressive neurodevelopmental disorder. Finally, Uc.309 expression was identified to be negatively correlated with the expression of an intron in *Btrc*, which modulates neural differentiation by degrading REST [9].

### Cardiovascular system

The cardiovascular system is critical for the maintenance of a normal physiological state by supplying oxygen and nutrients to all cells in the body while also removing waste and carbon dioxide. Many studies have identified a role of ucRNAs in normal cardiovascular development and function.

A study found the ucRNA, T-uc.167, which is located on the antisense chain of the coding gene *Mef2c,* exhibits high expression in patients with ventricular septal defect (VSD). Uc.167 leads to G1 arrest while uc.40 prolongs S phase to reduce cell proliferation and differentiation and to induce apoptosis. In this mechanism, *Mef2c* is negatively regulated by uc.167, however, overexpression of *Mef2c* can reverse the effects of uc.167 [10]. Uc.40 also has a known role in inhibiting the proliferation and differentiation of cardiomyocytes by downregulating Pre-B-cell leukemia homeobox transcription factor 1 (PBX1) expression [11]. Interestingly, although uc.245 has no direct effects on the cell cycle, it can play the same role as the ucRNAs discussed above by downregulating the expression of friend of GATA 2 (FOG2). In addition, the co-silencing of uc.245 and FOG2 can upregulate the expression of specific genes and proteins required for myocardial formation, thus promoting the differentiation of P19 cells into cardiomyocytes [12]. Similar to uc.167, a study found that uc.4 is located in the *Casz1* gene region but is expressed independently of this gene. Uc.4 does not affect cell proliferation and apoptosis, but its overexpression can inhibit the differentiation of P19 cells, which may have a negative impact on the development of cardiac structure and function [13].

### Other systems

In lead induced renal tubular epithelial cells, it was found that the upregulation of uc.173 significantly promotes the viability of Human proximal tubular epithelial cell line (HK-2) and human kidney proximal tubular epithelial cell line (HKC) exposed to lead, and also restores the expression of apoptosis related genes such as Caspase-3, Caspase-9, Cleaved Caspase-3 and Cleaved Caspase-9. But the function of T-UCR in lead-induced nephrotoxicity is still unknown [14]. This ucRNA also has known roles in the function of the intestinal epithelial barrier. Uc.173 acts as a natural decoy RNA for miR-29b, which directly interacts with claudin-1 (CLDN1) mRNA via the 3' untranslated region and represses its translation. Normal expression of CLDN1 restores the intestinal epithelial barrier function [15]. In anouther study, it was found that uc.291 interacts with actin-like 6A (ACTL6A) to modulate chromatin remodeling in keratinocytes in the basal layer of the epidermis, therefore allowing the transcription of late differentiation genes of the epidermis. The decrease of uc.291 expression enhances the ability of ACTL6A to bind to the promoters of Brahma homologue/Brahma-related gene 1 (BRM/BRG1), and subsequently downregulates the expression of *Edc*. This mechanism inhibits the activation of epidermal differentiation genes. Therefore, uc.291 is important for the switch between the epidermal progenitor state and differentiation into mature epidermal cells [16].

## Pathology

Disease is an extremely complex state in the body and can take many different forms. Interactions between pathogenic factors and the immune system can have dramatic impacts on cell and tissue morphology, metabolic states, and physiological function. Recent studies have identified potential roles of ucRNAs in the development of pathological disease states in various body systems(as listed in Table 1).

**Table 1:** Roles of ucRNAs in Pathology

ucRNA	Function	References
uc.360 +	Upregulate co-expression of P2X4 and active ERK1/2 signaling pathway to damage cardiac sympathetic nerve	[27]
uc.48 +	Upregulate P2X7, P2X3, P2Y12 receptor, downregulated p38 MAPK activity, release endogenous inflammatory mediators and promote the phosphorylation of ERK1/2 to regulate mechanical hyperalgesia and pain transmission in TN, HIV and diabetes	[17–23]
uc.98	Promote the development and vulnerability of atherosclerotic plaque by promoting adhesion and inflammatory response	[24]
uc.457	Inhibit the proliferation and differentiation of P19 cells	[25]
uc.323	Inhibit phosphorylation of ERK1/2, complex with mTORC1 and EZH2 to inhibit the transcriptional expression of CPT1b promoter to regulate H3K27me3 to prevent cardiomyocyte hypertrophy	[26]
uc.48 +	Interact with NR\U105053 and regulate EP300 to promote the bactericidal ability of macrophages and inhibit the intracellular reproduction of MTB	[28]
uc.372	Bind miR-195/mir-4668 and inhibit the maturation of it to promote liver fat production	[29]
uc.333	Negatively regulate miR-223 to alleviate insulin resistance (IR)	[30]
uc.261	Reduce TJP assembled on the cell membrane, causing occurrence of inflammatory response, damage of mucosal barrier and the increase of cell bypass permeability	[31, 32]
uc.412	Mediated by TGF-β1 and form a network with multiple signal pathways to cause mesangial proliferative nephropathy	[33]

### Neuroendocrine system

The development of neurological diseases is complex. The neurological impact of altered ucRNA expression caused by various diseases such as diabetes are summarized here. Diabetes may increase the expression of uc.360 + and uc.48 + and lead to neuropathy. Researchers found the silencing of these two ucRNAs could significantly reduce blood pressure, heart rate, and the ratio of low frequency to high frequency in heart rate variability, thus maintaining the dynamic balance between parasympathetic and sympathetic nerve activities [17, 18]. Mechanistically, they also found low expression of uc.360 + and uc.48 + can alleviate diabetic cardiac autonomic neuropathy (DCAN) and pain caused by diabetes and promote cardiac autonomic nerve remodeling by reducing the expression of P2X4, P2X3, and P2X7 respectively, thus resulting in the reduction of the level of inflammatory factors and inhibition of the ERK1/2 signaling pathway [18–21]. In addition, uc.48 + induces mechanical hyperalgesia and pain transmission of trigeminal neuralgia (TN) by increasing the expression of P2X7 [22]. Similarly, uc.48 + promotes human immunodeficiency virus (HIV) related neuropathic pain sensitivity by actively upregulating the P2Y12 receptor and downregulating p38 MAPK activity, thereby increasing intracellular cAMP concentration [23].

### Cardiovascular system

A properly functioning heart is as critical to the body as a properly functioning engine is to a car. However, cardiac pathologies are common and can have detrimental impacts on the life of effected individuals. Currently, cardiovascular diseases are the leading cause of death worldwide. The ucRNA, uc.98, was found to be involved in regulating the physiological state of endothelial cells and the stability of plaque. Uc.98 was shown to result in abnormal proliferation of aortic endothelial cells (AEC), enhanced adhesion, and to significantly increase the levels of inflammatory transcription factors by activating phosphatidylinositol 3-kinase/Akt/nuclear factor (NF)-κB pathway and increasing the expressions of ICAM-1 and VCAM-1, thus accelerating the process of atherosclerosis (AS) [24]. The overexpression of uc.457 can inhibit the proliferation of P19 cells in a time-dependent manner and reduce the expression of genes related to myocardial maturation such as Histone Cell Cycle Regulator (*HIRA*), Natriuretic Peptide A (*NPPA*), Troponin T2, Cardiac Type (*CTnT*) and Myocyte Enhancer Factor 2C (*Mef2c*), which demonstrates aberrant uc.457 expression can induce coronary heart disease [25]. A study found uc.323 could prevent myocardial hypertrophy by inhibiting the expression of atrial natriuretic peptide (ANP) and B-type natriuretic peptide (BNP). In addition, uc.323 can form a negative feedback loop with mTORC1 to inhibit the phosphorylation of ERK1/2, form a complex with enhancer of zeste homolog 2 (EZH2) to inhibit the transcriptional expression of the Carnitine Palmitoyl transferase 1b (CPT1B) promoter, and regulate trimethylation of lysine 27 of histone H3 (H3K27me3) to inhibit cardiomyocyte hypertrophy [26].

### Tumors

A tumor is an abnormal mass of tissue formed by local histiocytic hyperplasia under the action of various tumorigenic factors. Tumor development is a highly complex and multifactored process and can result in the formation of both benign or malignant tumors. Cancer results from the development of a malignant tumor which can spread both locally and systemically. Cancer is one of the leading causes of deaths worldwide and has no effective cure. A lot of studies have found that ucRNA plays an important role in cancer development (as listed in Table 2).

**Table 2:** Roles of ucRNAs in Tumors

ucRNA	Organ	Function	References
uc.338	Lung	Accelerate the invasion of lung cancer cells by promoting EMT related proteins	[34]
uc.339		Regulate uc.339/miR-339/SLC7A11 axis to promote lung adenocarcinoma	[35]
uc.83-		Regulate p-AKT and p-ERK1 to increase cell viability and promote cell growth	[36]
uc.454		Induce apoptosis of tumor cells by negatively regulating HSPA12B	[37]
uc.189	Esophagus	Inhibit the translation of EPHA2 and increase the phosphorylation of P38MAPK and VEGF-C to promote lymph node metastasis of tumor	[38, 39]
uc.160 +	Stomach Gila	Inhibit the phosphorylation of Akt by regulating PTEN and binding with miR-155 Promote the pro-apoptotic effect of RYBP and FOXP2 to regulate tumor suppressor genes, leading to a better prognosis	[3, 40, 41]
uc.63 +	Stomach Bladder Breast Prostate	Uc.63/NF-κB axis participates to develop GC Increase cell proliferation, inhibit apoptosis and induce CDDP resistance Inhibit cell death and promoting BC by increasing G2/M cells Positively regulates AR, resulting in the resistance of PC cells to docetaxel	[42, 50, 53, 54]
uc.134	Hepar	Inhibit HCC by accumulating CUL4A in the nucleus and increasing the Hippo kinase signal	[43]
uc.138	Colon	Promote colon cancer by increasing the percentage of G2/M cells and downregulating the genes related to cell death	[45]
uc.77-		Promote FBXW8 expression and ubiquitination of CDK4 to inhibit the proliferation of CRC cells	[46]
uc.416 + A	Kidney	Interact with miR-153 to negatively regulate EMT	[47]
uc.8 +	Bladder	Negatively regulate with miR-596 with DDX19B and NXF1 to lead to tumorigenesis and transport in the cytoplasm	[48, 49]
uc.147	Breast	Regulate surrounding proteins to increase the invasiveness of BC	[51]
uc.51		Regulate NONO protein, increase the phosphorylation of CREB and finally promote BC	[52]
uc.57		Downregulate BCL11A, inhibit P13K/AKT and MAPK signaling pathways to reduce TAM resistance	[56]
uc.38		Negatively regulate PBX1 to inhibit cell proliferation and promote apoptosis by regulating BCL-2 and BAX	[55]
uc.283 +	Gila	Negatively regulate Pri-miRNA interaction to promote gliomas	[3]
uc.291	Skin	Negatively regulate ACTL6A to reverse the inhibition of differentiation mediated by ACTL6A	[58]

In the respiratory system, uc.338 can accelerate the migration and invasion of lung cancer cells by promoting Epithelial–mesenchymal transition (EMT) related proteins, in which CYCLIN B1, CDC25C, Snail, vimentin and N-cadherin are all upregulated [34]. In another study, uc.339 promotes the development and metastasis of lung adenocarcinoma in vivo by regulating the uc.339/miR-339/SLC7A11 axis, where the inhibition of miR-339 leads to increased expression of SLC7A11 and weakens ferroptosis [35]. In addition, although uc.83- is fully located in the *Link01876* gene sequence, it can increase the number of cells in S-phase and promote the phosphorylation of p-AKT and p-ERK1 independently of *Link01876* gene expression. Through this mechanism, uc.83- acts to promote the development of small cell lung cancer [36]. The ucRNA, uc.454, can negatively regulate its direct target gene *Hspa12b* to induce apoptosis of tumor cells, therefore acting as a tumor suppressor [37].

In esophageal carcinoma, uc.189 is transferred from esophageal squamous cell carcinomas (ESCC) to human lymphatic endothelial cells (HLECs) through exosomes, allowing ESCC cells to metastasize to distant locations through lymphatic vessels. In addition, uc.189 inhibits the translation of EPHA2 and increases the phosphorylation of P38MAPK and VEGF-C at the mRNA and protein levels, thereby promoting lymph angiogenesis and tumor lymphatic metastasis [38, 39].

In gastric cancer (GC), uc.160 + may inhibit the phosphorylation of AKT and the activation of MAPK signaling by regulating PTEN, thus inhibiting the proliferation, apoptosis, and viability of GC cells [40]. In another study, uc.160 + was found to bind directly with miR-155 to inhibit the growth of GC. Although there is a negative correlation between them, both act as tumor suppressors. This inconsistency warrants exploration into the role of uc.160 + in GC [41]. Conversely, overexpression of uc.63 + can induce p65 activity in the NF-κB complex leading to cell proliferation [42].

In the occurrence and development of hepatocellular carcinoma (HCC), the persistent low expression of uc.134, located in the intron region of *Rsrc1,* may lead to the low survival rate of HCC patients. Uc.134 can inhibit the development of HCC by inhibiting Cullin 4A (CUL4A) ubiquitination of Large Tumor Suppressor Kinase 1 (LATS1) and increasing YAPS127 phosphorylation, thereby activating the Hippo kinase signal [43]. In another study on liver cancer, it was found that the overexpression of uc.306 may lead to the further development of liver cancer. Uc.306 may be involved in EMT via BTRC (β-TrCP)-Wnt pathway, but the specific mechanism is not clear [44].

In recent years, the incidence rate of colorectal cancer has risen sharply. Therefore, studying the roles of ucRNAs in colorectal cancer is imperative. Uc.138 is located on chromosome 3 of *Tra2b*. It was identified that overexpression of uc.138 can increase cell proliferation by increasing G2 and S phases-related gene expression, including CDK1, cyclin A, and cyclin B and by downregulating genes related to cell death to reduce apoptosis. Consequently, this results in the resistance of colon cancer cells to anticancer drugs and promotes the migration of tumor cells [45]. In another study, uc.77- inhibited the growth of colorectal cancer (CRC) cells by blocking the G0/G1 phase through downregulation of the CDK4 protein (affected by E3 ligase). Interestingly, uc.77- can compete with F-Box and WD Repeat Domain Containing 8 (FBXW8) mRNA to bind to miR-4676-5p, thus promoting FBXW8 expression and ubiquitination of CDK4 to inhibit the proliferation of CRC cells [46].

In the urinary system, uc.416 + A, which is associated with renal cell carcinoma, and uc.8 + , which is associated with bladder cancer, negatively regulate their corresponding miRNAs to stimulate EMT and tumorigenesis [47, 48]. However, in contrast to uc.416 + A expression in renal cell carcinoma, the expression of uc.8 + is negatively correlated with the grade of bladder cancer by increasing the expression of matrix metalloproteinase 9 (MMP9). Another study on uc.8 + in the context of bladder cancer found it’s not only expressed in the early stage of bladder cancer, but also in the tumor microenvironment. The interaction network formed by DEAD-box helicase 19B (DDX19B) and nuclear RNA export factor 1 (NXF1) may participate in the transport of uc.8 + to the cytoplasm [49]. Uc.63 + induces androgen receptor (AR) expression in UMUC3 cells at the mRNA and protein levels, resulting in cisplatin (CDDP) resistance. Based on this finding, a study identified knockdown of uc.63 + can re-sensitize CDDP-resistant UMUC3 cells to drugs used for bladder cancer treatment [50].

Breast cancer (BC) is the most common cancer among women worldwide. The carcinogenic ucRNA, uc.147, resides in the 51st intron of the gene *Lrba*. *Tacr3* and *Cxxc4*, which are positively correlated with their adjacent genes, were found to be related to estrogen receptors. Moreover, the combination of *Cxxc4* and uc.147 can inhibit the expression of *Cxxc4* and increase the invasiveness of tumors [51]. Uc.51 is also a carcinogenic ucRNA which acts at the transcriptional level by stably and positively regulating non-POU domain-containing octamer-binding protein (NONO) and increasing the phosphorylation of CREB [52]. Similarly, uc.63 can increase G2/M cells, inhibit cellular apoptosis, and promote BC and prostate cancer (PC) independently of the *exportin-1* gene (*Xpo1*) [53]. Uc.63 + positively regulates AR, which leads to the resistance of prostate cancer cells to docetaxel. In addition, uc.63 + can also downregulate the expression of miR-130b of MMP2, thus playing a carcinogenic role [54]. Unlike the ucRNAs mentioned above, uc.38 is a tumor suppressor. The gene *Pbx1* is negatively regulated as a downstream target gene of uc.38, which promotes apoptosis and inhibits proliferation by reducing BCL-2 expression and increasing BAX expression [55]. In the treatment of BC, Western blot analysis identified uc.57 can downregulate the expression of BCL11A at both the mRNA and protein level. It also acts to inhibit P13K/AKT and MAPK signaling pathways and subsequently reduce tamoxifen (TAM) resistance [56].

In central nervous system tumors, a study identified uc.283 + was overexpressed in gliomas while uc.160 + was expressed at a low level. However, the methylation of uc.160 + related CpG islands can promote the proapoptotic effect of RING1 and YY1-binding protein (RYBP) targets and forkhead box P2 (FOXP2) to regulate tumor suppressor genes and proto-oncogenes by regulating and editing pri-miR-376 clusters, thus leading to a better prognosis of glioma patients [57]. Conversely, uc.283 can negatively regulate ncRNA interaction to prevent pri-miRNA-195 from cleaving by Drosha [3].

Finally, ucRNAs also have identified roles in metastatic and fatal cutaneous squamous cell carcinoma (CSCC) derived skin cancer. The gene *ACTL6A* was found to regulate the SWI/SNF chromatin remodeling complex as a carcinogen in squamous cell carcinoma to maintain the tumor in an undifferentiated state. Interestingly, uc.291 can act negatively on *ACTL6A*, reversing the inhibition of differentiation genes mediated by *ACTL6A* [58].

## Discussion

**Fig. 2:**
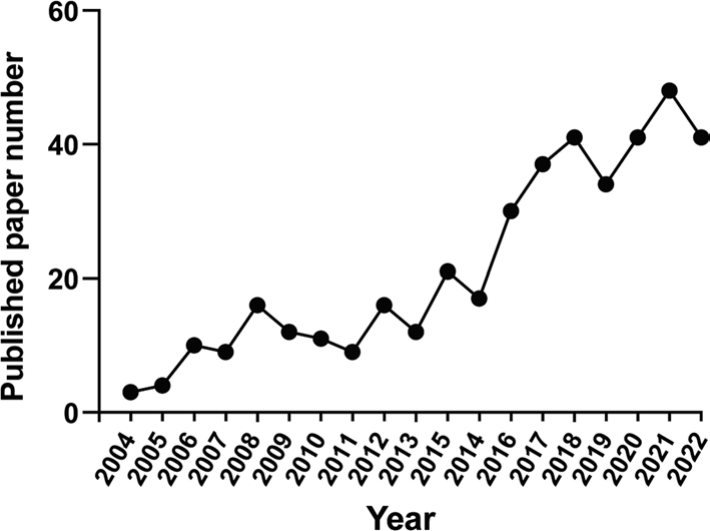
The publications related to ucRNAs in recent years. The number of papers related to ucRNA research is presented since the discovery of the ultra-conserved region in 2004. As can be seen from the figure, the number of papers generally maintains an upward trend, which means that ucRNA is attracting more and more attention and is worthy of further study

It is known that ultra-conserved RNA is conserved among humans, mice, and rats, which suggests ucRNAs are highly conserved among species. Not only ucRNAs are conserved among species, but they are also evolutionarily conserved indicating a critical developmental and/or physiological importance of these molecules. Therefore, research on ucRNAs has become increasingly prominent in recent years (as shown in Fig. 2). Despite increased research attention, ucRNA research is still in a stage of infancy compared to other biological molecules, such as DNA and proteins. Therefore, it is necessary to summarize the current understanding of ucRNAs and to prompt further research.

In terms of application prospects, ucRNA have the potential to be used as diagnostic markers and novel therapeutic targets for the treatment of various clinical diseases. For example, many ucRNAs can act as magnets to adsorb microRNAs, thus competing for binding sites with microRNAs. This results in negative interaction with the ucRNA’s corresponding microRNA and ultimately influences the occurrence and development of tumors. Similar mechanisms may also occur to facilitate the regulation of physiological and pathological changes in the human nervous system, digestive system, urinary system, and many other body systems. Mechanistically, ucRNAs also play a key role in the development of neuropathic pain by regulating ion channels and neuroinflammation. For example, activation of the P2X receptor by ucRNA promotes phosphorylation of ERK1/2 and the release of pro-inflammatory factors, which promote pain transmission and exacerbate mechanical and thermal hyperalgesia caused by various diseases. However, currently, the P2X receptor has only been shown to be involved in trigeminal neuralgia and pain caused by diabetes and acquired immunodeficiency syndrome (AIDS). Therefore, further study on the impact of this pathway in other types of pain, such as pain caused by cancer is warranted.

Although our understanding of the functions of ucRNA over the past two decades has dramatically increased, the application of ucRNA in disease treatment still faces many challenges. Thus, the development of ucRNA treatment methods is still in the pre-clinical or basic research phase. Translation from basic and clinical research to direct clinical applications that can alleviate disease burden requires increased research into the properties of these molecules. Compared with the stable properties of DNA and matured technologies associated with protein studies, RNA is easily degraded and damaged, and technological methods are unsatisfactory. In addition, much effort is still required to improve the quality of ucRNA research to more accurately and efficiently explore the specific intermolecular mechanisms of ucRNA functions. Future advancement in technology and research efforts will significantly expand the understanding of both the specific molecular signaling mechanisms of ucRNAs and the specific pedigree relationships with other RNAs and/or proteins. Using current knowledge and experience to study other RNAs in the lncRNA family, such as microRNAs, will provide a critical reference point to expand ucRNA research strategies. These studies will be invaluable for the developmental success of efficient and feasible protocols for ucRNAs to be implemented as clinical therapeutics.

## Data Availability

Data sharing is not applicable to this article as no new data were created or analyzed in this study.
